# Prevention of enamel demineralization with a novel fluoride strip: enamel surface composition and depth profile

**DOI:** 10.1038/srep13352

**Published:** 2015-08-21

**Authors:** Bor-Shiunn Lee, Po-Hung Chou, Shu-Yu Chen, Hua-Yang Liao, Che-Chen Chang

**Affiliations:** 1 Graduate Institute of Oral Biology, School of Dentistry, National Taiwan University and National Taiwan University Hospital, No.1, Changde St., Jhongjheng District, Taipei 100, Taiwan; 2 Department of Chemistry, National Taiwan University, No. 1, Sec. 4, Roosevelt Road, Taipei 10617, Taiwan; 3 Research Center for Applied Sciences, Academia Sinica, 128 Academia Road, Section 2, Nankang, Taipei 11529, Taiwan

## Abstract

There is no topically applicable low concentration fluoride delivery device available for caries prevention. This study was aimed to assess the use of a low concentration (1450 ppm) fluoride strip as an effective fluoride delivery system against enamel demineralization. The enamel surface composition and calcium-deficient hydroxyapatite or toothpaste treatments were investigated using X-ray photoelectron spectroscopy. *In vitro* enamel demineralization was assayed using a pH cycling model and the dissolution of calcium ions from the treated specimens was quantified using ion chromatography. After 24-hr fluoride-strip treatment, the enamel was covered with a CaF_2_ layer which showed a granular morphology of 1 μm in size. Below the CaF_2_ layer was a region of mixed fluorapatite and CaF_2_. Fluoride infiltrated extensively in enamel to produce highly fluorinated fluorohydroxyapatite. In comparison, low-fluoride-level fluorinated fluorohydroxyapatite was formed on the enamel specimen exposed to toothpaste. The treatments with the fluoride strip as short as 1 hr significantly inhibited enamel demineralization. The fluoride strip was effective for topical fluoride delivery and inhibited *in vitro* demineralization of enamel by forming CaF_2_ and fluoride-containing apatites at the enamel surface. It exhibited the potential as an effective fluoride delivery device for general use in prevention of caries.

Dental caries is one of the major causes of dentition loss and oral health impairment. Fluorides have been well recognized as caries preventive agents to inhibit demineralization and promote remineralization of dental hard tissues^1^,^2^,^3^,^4^. The mechanisms of fluoride anticariogenicity include the inhibition of demineralization and the enhancement of remineralization^5^. Interactions of fluoride with dental hard tissues can produce a more stable acid-resistant hydroxyapatite (HAP) lattice^6^. The reaction products after fluoridation have been reported to include calcium fluoride (CaF_2_), calcium hydroxide (Ca(OH)_2_), and fluorapatite (FAP, Ca_5_(PO_4_)_3_F)^7^,^8^. The fluoride-containing compounds can protect the enamel surface and serve as fluoride reservoirs.

Topical fluoride delivery for caries prevention includes fluoride rinses, fluoridated toothpastes, topical fluoride gels and solutions, and fluoride varnishes^9^. Fluoridated toothpastes are the most cost-effective and widely used products among the topical methods^10^. However, human salivary fluoride clearance studies showed that fluoride concentration decreased in two distinct phases: an initial rapid increase of topical fluoride clearance that lasted for 40–80 min and a second slowly declining phase of fluoride release from oral fluoride reservoirs that lasted for several hours^11^. Other commonly used topical fluoride agents are acidulated phosphate fluoride containing 1.23% fluoride in the form of sodium fluoride at pH 3.5, and fluoride varnishes like Duraphat containing 5 wt% sodium fluoride or 2.26 wt% F^−^ at pH 4.5 in an alcoholic suspension of natural resins^12^. For effective treatments, both agents have to be applied at high concentrations in-office by professionals and their long-term use is inconvenient. Recently, casein phosphopeptide-amorphous calcium phosphate complexes have been developed and have been added to chewing gum, mouth-rinse, sealants, and milk for enhanced remineralization of enamel^13^,^14^. Their long-term efficacy in caries prevention needs to be further investigated.

Slow-release devices of low level fluoride are regarded as a potential method to elevate fluoride concentration at the biofilm/saliva/dental interface and inhibit dental caries^15^,^16^. These developed devices include copolymer membrane devices, glass devices, and the hydroxyapatite-Eudragit RS100 diffusion controlled F system^17^. We developed fluoride-releasing strips for caries prevention (certification number I371287 of the Taiwan Patent Office) and hypothesized that use of a low concentration (1450 ppm) acidulated gel-based fluoride application strip would be effective for fluoride delivery to the enamel surface for caries prevention. Using X-ray photoelectron spectroscopy (XPS) and scanning electron microscopy (SEM), we examined the nature of the fluoride deposition at the enamel surface and also investigated the potential caries preventive action of the fluoride strip application using an *in vitro* enamel demineralization assay.

## Materials and Methods

### Specimen preparation

Glycerol was added to deionized water to form a 5 wt% solution. Fluoride gel with a concentration of 1450 ppm was prepared by dissolving 0.32 g NaF in 100 g solution, adjusting the pH to 5.5 with phosphoric acid and adding 2.5 wt% carboxymethyl cellulose to increase viscosity. The mixture was stirred for 12 h and subsequently applied to a polyvinylchloride membrane and cast to a predetermined 500-μm thickness using a Gardener knife. The membrane was used as the vehicle to produce fluoride strips. Because slow-release devices of low levels of fluoride were not available in commercial products of Taiwan, Colgate toothpaste (1450 ppm, Colgate-Palmolive CO., NY, USA) was used for comparison. The toothpaste was also applied to a polyvinylchloride membrane and prepared to a predetermined 500-μm thickness using a Gardener knife.

Extracted human permanent molars from people aged 16 to 40 years were used after obtaining informed consent from the donors. Permission to collect human teeth was obtained from the Ethical Committee of National Taiwan University Hospital (Case No. 201105080RC). Crowns with caries, restorations, or fractures were discarded. Any remaining soft tissues were thoroughly removed from the tooth surfaces with a dental scaler (Sonicflex 2000, KaVo Co., Biberbach, Germany) under running water. All teeth were stored until use in 4 ^o^C distilled water containing 0.2% thymol to inhibit microbial growth.

While fully hydrated, an undamaged enamel surface was prepared (3 mm ×3 mm in size, 1 mm in thickness) by means of a low-speed diamond wafering blade (Isomet; 10.2 cm ×0.3 mm, arbor size 1/2 inch, series 15HC diamond; Buehler Ltd., Lake Bluff, IL) and the root portions were discarded. Flat enamel surfaces were created by wet grinding each specimen on a polishing machine with 240 grit and then 600 grit SiC papers (Buehler Ecomet V, Buehler Ltd., Lake Bluff, IL, USA) for 30 s each. All surfaces of each specimen were coated with epoxy resin except the outer enamel surface, which would receive treatment.

### Lesion formation, pH cycling conditions, and baseline calcium measurements

**Figure 1:**
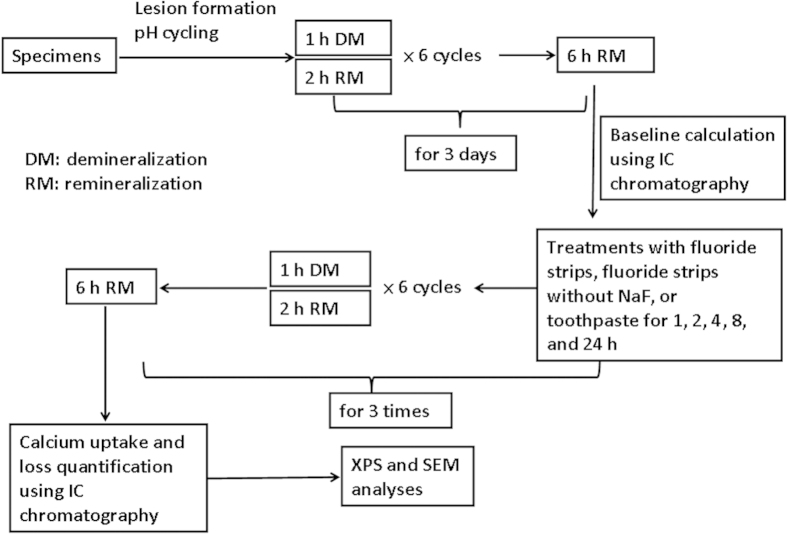
Flowchart of the experimental design.

Shallow lesions were formed on the enamel specimens in a two-layer system of 8% methyl cellulose gel and 0.1 M lactic acid adjusted to pH 4.6 for 7 days^18^. The enamel specimens were submitted to pH cycles including demineralizing, rinse, remineralizing, and rinse solutions for 3 days at 37 ^o^C^19^. Each specimen was cycled in 4 ml of the three solutions. Six 3-hour cycles each day were programmed with 1 h in demineralizing solution containing 1.5 mM CaCl_2_, 0.9 mM KH_2_PO_4_ and 50 mM acetic acid adjusted to pH 4.8. They were then stored in rinse solution containing 1.5 mM CaCl_2_, 0.9 mM KH_2_PO_4_, 130 mM KCl at pH 7.0 for 5 s. Subsequently, the specimens were immersed in a remineralizing solution containing 1.5 mM CaCl_2_, 0.9 mM KH_2_PO_4_, 130 mM KCl, and 20 mM HEPES, pH 7.0 for 2 h. They were then rinsed again as described before. The specimens were immersed in remineralization solution for the remaining 6 h (Fig. 1). The initial 3 days of pH cycling were used to calculate the baseline calcium uptake and loss of each specimen^20^.

Calcium uptake and loss were analyzed by sampling the de- and re-mineralizing solutions for changes in the calcium content using a Metrohm Compact ion chromatograph (IC) 861 Chromatography instrument with a conductivity detector and a 100 μl injection loop. The separation column was a Metrohm IC anion column Metrosep A Supp5, 150 × 4.0 mm, packed with polyvinyl alcohol with quartenary ammonium groups, and was used with a Metrohm IC precolumn A Supp 4/5 Guard. The eluent flow-rate was 0.7 ml/min (sodium hydrogen carbonate 168 mg/2 L and sodium carbonate 678 mg/2 L). A stock solution of calcium was prepared by dissolving appropriate amounts of analytical reagent grade calcium salts in high purity water. The solution was then diluted for calibration.

### Fluoride strips or toothpaste treatments and calcium measurements

Two hundred and twenty-five human enamel specimens were randomly assigned to 3 groups (n = 75) for receiving treatments with either fluoride strips, fluoride strips without NaF, or toothpaste after 3 days of pH cycling. Each treatment group was further divided into 5 subgroups (n = 15) for different treatment times (1 h, 2 h, 4 h, 8 h, and 24 h). Specimens received only lesion formation and pH cycling for 3 days (n = 15) were used as the control. Treatments with fluoride strips, fluoride strips without NaF, or toothpaste were done after each of the 6-hour periods (Fig. 1) in a 37 ^o^C humid environment. After the treatments, specimens were rinsed with deionized water for 1 min, tapped dry, and returned to pH cycling. Each treatment and the following pH cycling were performed 3 times. After the final pH cycling, the specimens were rinsed again with deionized water for 1 min. Calcium uptake and loss were quantified using IC for changes in the calcium content (baseline vs. after treatment in the de- and re-mineralizing solutions).

### XPS analysis

After calcium measurements using IC, specimens were examined using XPS (Thermo Scientific, UK) (n = 3) and SEM (Model S-3500N, Hitachi, Japan) (n = 3). Calcium fluoride (Sigma-Aldrich, purity ≥ 99.5%), hydroxyapatite (Sigma-Aldrich, purity ≥ 99.5%), human enamel, and human enamel receiving only lesion formation and pH cycling for 3 days were also examined for references and comparison.

Treated specimens for different exposure times and ion-etching depths were studied using ESCALAB 250 and K-alpha XPS systems, respectively, with monochromatic Al Kα X-rays (beam energy = 1486.6 eV). The X-ray sources were operated at ~200 W. The system operating pressures were ~1 × 10^−9^ torr for exposure experiments and ~5 × 10^−8^ torr for depth profiling after the specimens were dehydrated at room temperature in a low-pressure chamber of less than 0.1 torr for ~1 day and parked in the preparation chamber for 2–3 days. High resolution spectra of fluorine (F 1s) and calcium (Ca 2p) were obtained over the respective binding energy ranges using pass energies of 20 eV for exposure experiments and 50 eV for depth profiling at 0.1 eV/step each. All XPS spectra were taken with the use of an electron flood gun for charge neutralization. The binding energy was calibrated with respect to C1s core level of adventitious carbon at 284.6 eV. For XPS depth profiling, an Ar ion source with ion energy of 3000 eV was used and the spectra were collected every 5 ~ 15 min. In the determination of the peak area, a Shirley-type background between two points that were 6 eV from the nearest peaks were chosen to be subtracted from each spectrum to decrease the inaccuracy in quantification^21^.

### SEM observation

Specimens receiving the fluoride strip treatment for 24 h plus pH cycling (n = 3) or the toothpaste treatment for 24 h plus pH cycling (n = 3) were further treated with 10 ml of 1 M KOH for 24 h, rinsed with 200 ml of distilled water for 5 min, and wiped with tissue paper^22^. These specimens and the ones after IC measurements were mounted on aluminum stubs, sputter-coated with ~20 nm thick gold/palladium and finally examined at 50000× magnification using SEM with an accelerating voltage of 15 kV.

### Statistical analysis

Statistical analysis was performed using the Statistical Package for Social Science (SPSS, Version 12.0, Sinter Information Group, Taipei, Taiwan). Tests were 2-tailed with a significance level of 0.05. Descriptive statistics for continuous variables were calculated and reported as the mean ± standard deviation (SD). The data were analyzed using one-way analysis of variance (ANOVA). If the data matched the assumption of normality and homogeneity of variance, Fisher’s multiple comparison tests provided a follow-up comparison among the three above-mentioned treatments (fluoride strips, strips without NaF, or toothpaste treatments).

## Results

**Figure 2:**
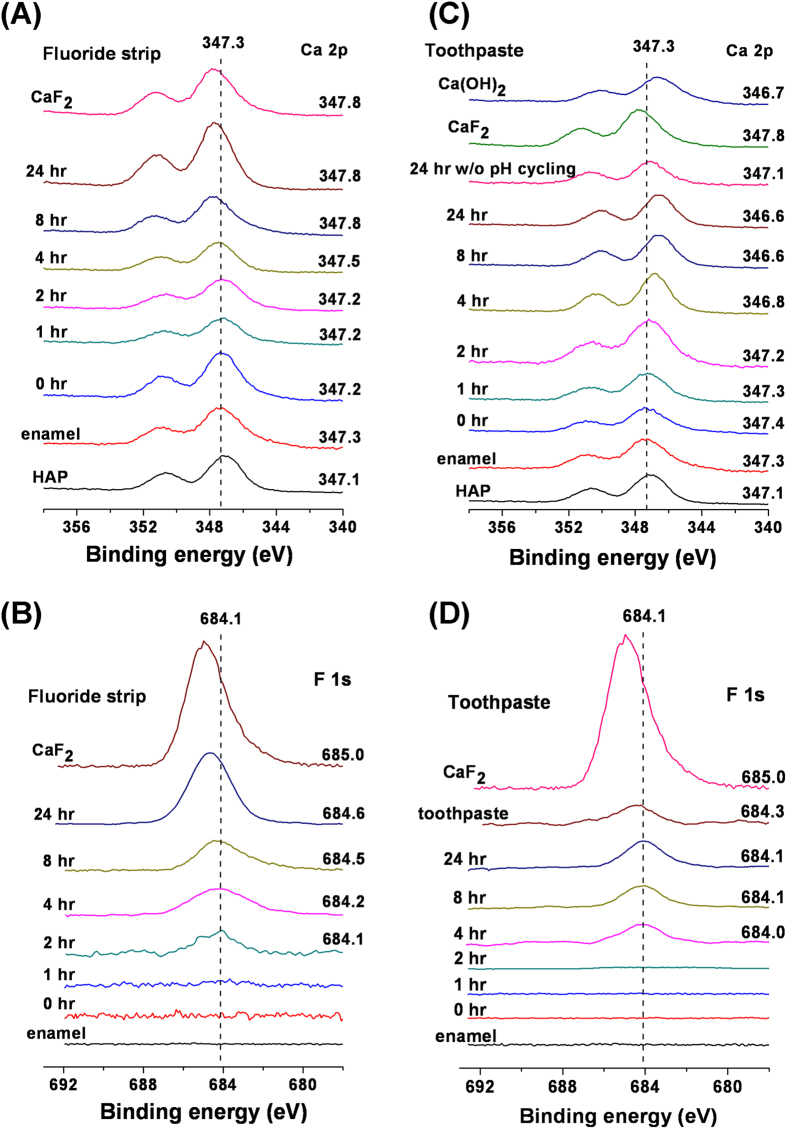
(**A,C**) Ca 2p and (**B,D**) F 1s XPS spectra taken from the specimen surfaces following exposure to (**A,B**) the fluoride strip and (**C,D**) toothpaste for the specified periods of time and then pH cycling. Their related Ca 2p and F 1s spectra taken from HAP, the pristine enamel, Ca(OH)_2_, and CaF_2_ are included in respective plots for comparison.

Figure 2 shows the XPS spectra of enamel exposed to the fluoride strip or toothpaste for different periods of time. No peaks were observed in the F 1s spectrum of the enamel specimen before the exposure (Fig. 2(B,D)). However, when the enamel specimen and hydroxyapatite were placed side-by-side on the sample holder, the Ca 2p peak of the enamel specimen persistently had a binding energy slightly higher than that of hydroxyapatite (Fig. 2(A,C)). For the fluoride strip group, the F 1s signal was detected when the exposure time was above 2 hr (Fig. 2(B)). Its peak position shifted to higher binding energy as the exposure time was increased. In addition, the Ca 2p peak also gradually shifted to higher binding energy as the exposure time was increased to 8 hr (Fig. 2(A)), after which the peak position remained almost unvarying at 347.8 eV. For the toothpaste group, the F 1s signal was detected when the treatment time was above 4 h (Fig. 2(D)). As the treatment time was increased, the F 1s peak was positioned almost invariably, in contrast to the shift of the F 1s signal observed from the fluoride-gel-treated specimen. The F 1s signal intensity was smaller than the one detected at the same exposure time from the fluoride-gel-treated specimen (Fig. 2(B,D)). The rate of the F 1s signal increase with exposure time was also lower than that observed from the fluoride-gel-treated specimen. In addition, the Ca 2p peak gradually shifted to lower binding energy as the treatment time was increased to 8 hr (Fig. 2(C)), after which the peak position remained almost unvarying at 346.6 eV.

**Figure 3:**
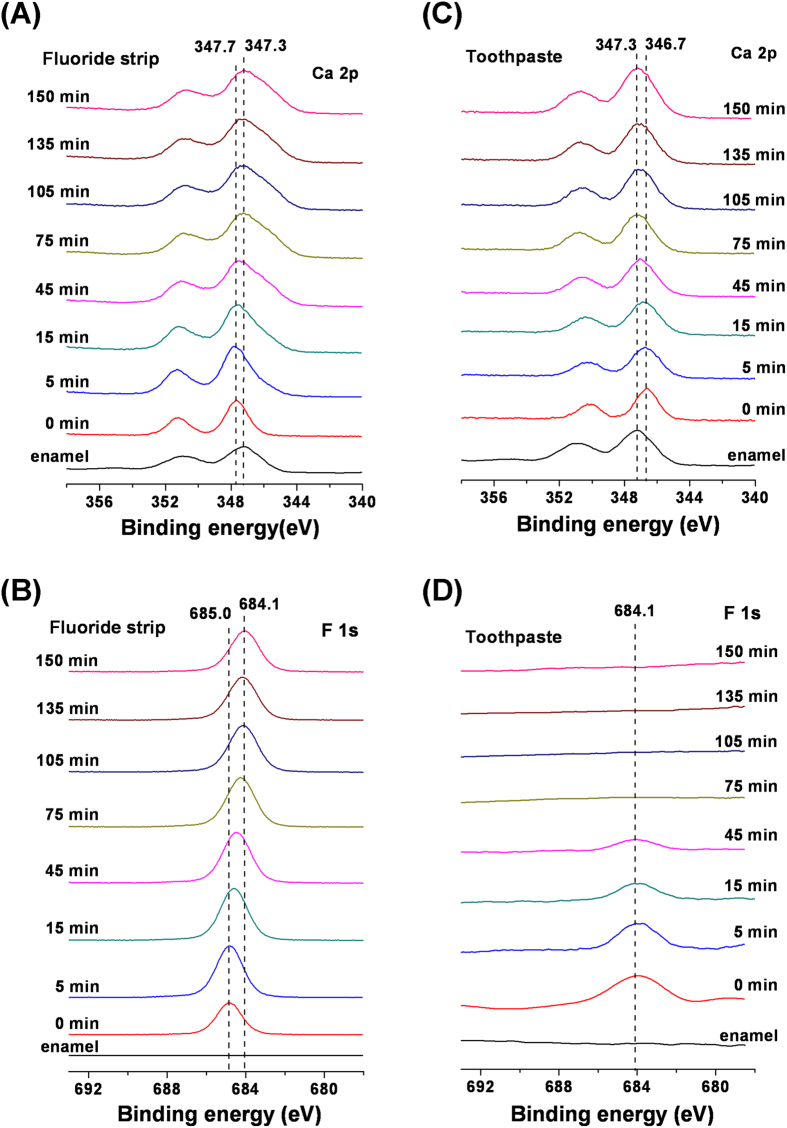
(**A,C**) Ca 2p and (**B,D**) F 1s XPS spectra taken from the 24-hr, (**A,B**) fluoride-strip- and (**C,D**) toothpaste-treated specimen surfaces following ion etching for the specified periods of time. Their related Ca 2p and F 1s spectra taken from the pristine enamel are included in respective plots for comparison.

The XPS depth profiling of the enamel treated with the fluoride strip or toothpaste for 24 hrs is shown in Fig. 3. For the fluoride strip group, the Ca 2p peak shifted into the direction of lower binding energy, in addition to the peak broadening due to differential surface charging induced by the sputtering from ion etching, as the time under ion etching increased (Fig. 3(A)). It shifted almost back to the Ca 2p peak position of enamel after etching for 75 min. However, the F 1s peak was still detected after ion etching for 150 min (Fig. 3(B)). For the toothpaste group, the Ca 2p peak shifted with ion etching time into the direction of higher binding energy, as the time under ion etching increased (Fig. 3(C)). It also shifted almost back to the Ca 2p peak position of enamel after ion etching for 75 min. The F 1s signal was detected up to the ion etching time of 45 min only (Fig. 3(D)).

**Figure 4:**
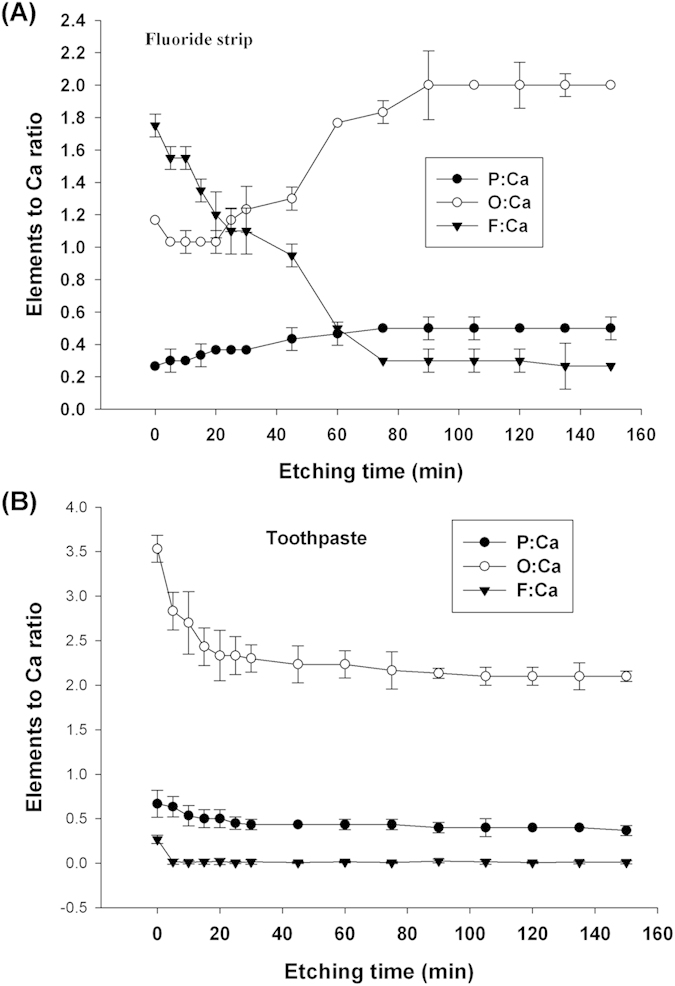
XPS depth profile of P 2p, O 1s, and F 1s for 24-hrs: (**A**) fluoride-strip- and (**B**) toothpaste-treated specimens, atomic ratio (to Ca) vs. sputtering time

Changes in atomic ratios for O/Ca, F/Ca, and P/Ca with ion etching time were observed in XPS depth profiling for the specimens receiving the fluoride-strip or toothpaste treatment (Fig. 4). After the fluoride-strip treatment for 24 h, the F/Ca ratio measured at 0 min of ion etching reached a high value of 1.75 (Fig. 4(A)). The ratio then gradually decreased to 0.30 after 75 min of ion etching and all three ratios became stable after 90 min of ion etching. After the toothpaste treatment for 24 h, the measured F/Ca ratio at 0 min of ion etching reached a value of 0.27 (Fig. 4(B)). It drastically decreased to ~0 within 5 min of ion etching and became almost unvaried with etching time thereafter. The initial O/Ca ratio was as high as 3.53. The O/Ca ratio dropped to 2.3 within 30 min of ion etching. It further decreased to the level of ~2.1 after 90 min of etching.

Results of the *in vitro* enamel dissolution assay demonstrated that the resistance of the enamel surface to demineralization increased in proportion to the fluoride strip treatment time (Table 1). For the specimens exposed to strips not containing NaF, IC assays demonstrated similar calcium loss and net calcium values irrespective of treatment time, indicating that the exposure exhibited no effect on enamel demineralization or remineralization. Compared with the toothpaste treatment, the specimens undergoing the treatment by the fluoride strip exhibited less calcium loss and higher calcium uptake for all treatment periods. Significant differences were found between these two treatments for net calcium values.

**Figure 5:**
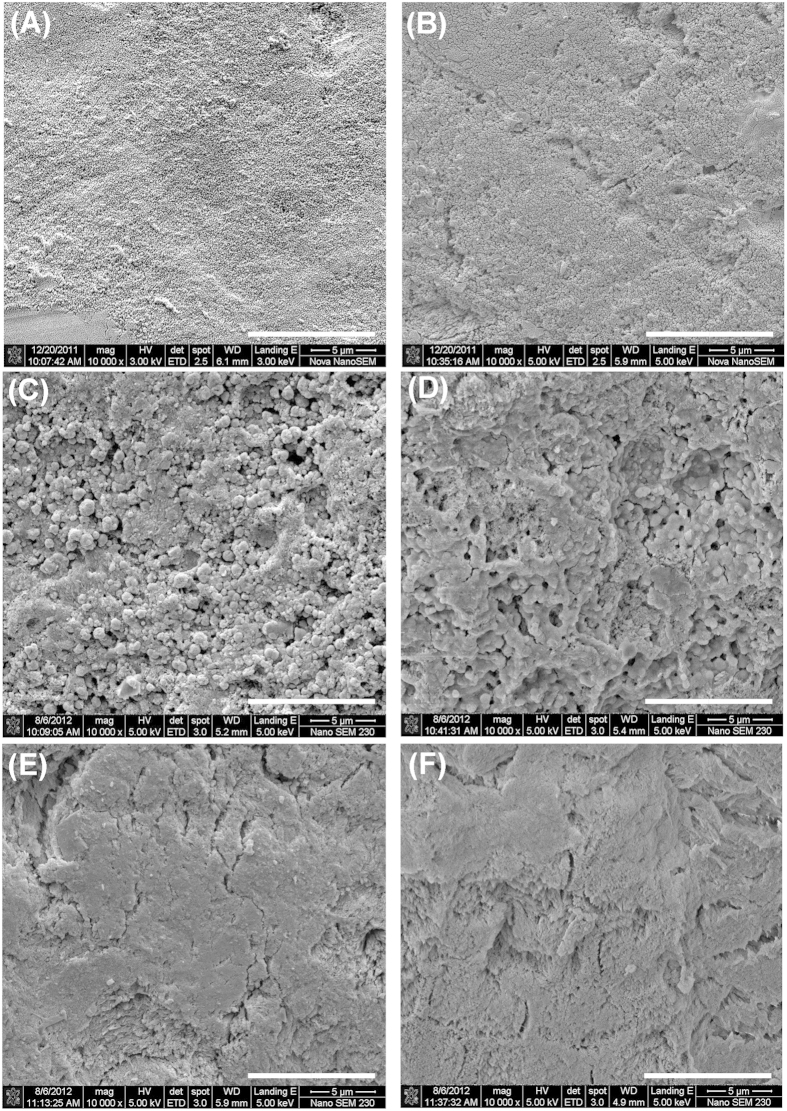
SEM images obtained from the enamel specimens (**A**) before and (**B**) after pH cycling. Dense granular structures were observed after fluoride-strip treatment for 24 h (**C**) and the granular structures diminished to some extent after KOH treatment (**D**). No particular morphology (like granular structures) was found after toothpaste treatment for 24 h (**E**) and the morphology remained after KOH treatment (**F**). The white scale bar at the lower right-hand corner of each image represents 10 μm.

SEM showed that, prior to treatments, human enamel had a smooth surface without any prominent features (Fig. 5(A)). After pH cycling, the surface became slightly rough (Fig. 5(B)). Granular structures were found on the surface after the fluoride-strip treatment for 24 h (Fig. 5(C)). They were reduced to some extent after KOH treatment (Fig. 5(D)). In contrast, the enamel surface did not exhibit particular morphology like granular structures after toothpaste treatment for 24 h (Fig. 5(E)). The morphology did not change prominently after KOH treatment (Fig. 5(F)).

## Discussion

The enamel specimen contained fluoride, as suggested by the persistently higher binding energy of the Ca 2p peak obtained from the enamel specimen than that from the hydroxyapatite (Fig. 2(A,C)). The fluoride existed in the form of fluorohydroxyapatite (FHAP, Ca_10_(PO_4_)_6_(F,OH)_2_). Its level was very low such that no F 1s signal was detectable by XPS (Fig. 2(B,D)). The low-fluoride-level FHAP present on the initial surface of the enamel specimen (i.e., obtained after grinding and polishing) is termed LFHAP herein. For the fluoride strip group, the F 1s signal detected at exposure time of more than 2 hr (Fig. 2(B)) indicated that fluorination of LFHAP, took place. The fluorinated LFHAP present on the enamel specimen surface after experiments that gives a detectable F 1s signal by XPS is termed FHAP herein. As shown in Fig. 2(A,B), the apatite on the specimen surface gradually transformed to CaF_2_ (with Ca 2p_3/2_ at 347.8 eV) at increasing exposure time. The F/Ca ratio of 1.75 obtained at 0 min etching time in the XPS depth profiling study on the fluoride-gel-treated specimen (Fig. 4(A)) was also in close agreement with the presence of CaF_2_ (in which F/Ca was 2) on the surface. The surface morphology changes observed in Fig. 5(A–D) also supported the notion that CaF_2_ was the main surface component^23^ of the fluoride-strip-treated enamel.

For the toothpaste group, the transformation of LFHAP to CaF_2_ was negligible, as revealed by the almost unvaried F 1s peak position with exposure time (Fig. 2(D)). Instead, the transformation of LFHAP to FHAP occurred via replacement of OH^−^ in LFHAP by F^−^ from toothpaste. It resulted in the F/Ca ratio of 0.27 measured at 0 min of ion etching from the specimen exposed to toothpaste for 24 hr (Fig. 4(B)). A test on submitting the 24-hr-toothpaste-treated enamel specimen to cleaning by sonication in deionized water, instead of to pH cycling, showed that the Ca 2p peak shifted substantially less to lower binding energy (with Ca 2p_3/2_ at 347.1 eV for water cleaning vs. 346.6 eV for pH cycling, Fig. 2(C)). It indicated that the OH^−^ ions replaced by F^−^ were hydrogen-bonded to the F species at the surface^6^,^24^ such that at increasing exposure time, more slaked lime, Ca(OH)_2_ (with Ca 2p_3/2_ at 346.7 eV), was formed on the specimen surface (Fig. 2(C)) after OH^−^ interacting with Ca^2+^ during pH cycling. Both slaked-lime-containing hydroxyapatite (SHAP) and FHAP were thus the main surface components of the specimen after lengthy exposure to toothpaste.

**Figure 6:**
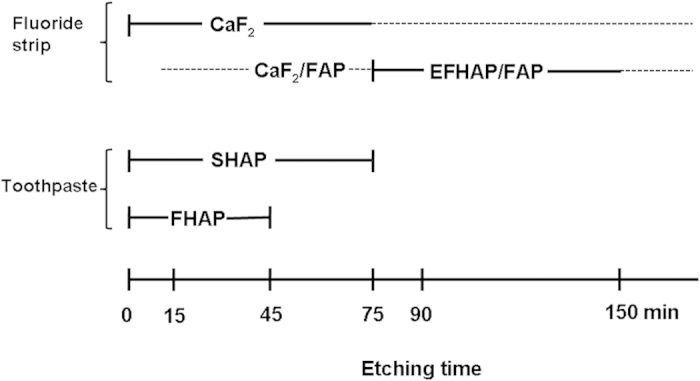
The chemical depth profile of enamel treated with a fluoride strip or toothpaste for 24 h. The dashed line represents predictions based on the data obtained.

FHAP’s fluoride content in the specimen exposed to toothpaste was less than that in the specimen exposed to the fluoride strip (Fig. 2(B,D)). The F/Ca ratio measured at 0 min etching time (Fig. 4(A,B)) indicated that, in comparison with the formation of FHAP in the toothpaste-treated specimen, extensive fluorinated FHAP (termed EFHAP) and FAP may be formed on the specimen exposed to the fluoride strip. The chemical depth profile in enamel treated with fluoride strip or toothpaste for 24 h is illustrated in Fig. 6.

Results obtained from the XPS depth profiling study supported the formation of CaF_2_, EFHAP, and FAP on the specimen exposed to the fluoride strip. As the time under ion etching increased, the observed shift of the Ca 2p peak to lower binding energy (Fig. 3(A)) was consistent with the removal of CaF_2_ from the fluoride-strip-treated specimen by the ion beam. After etching for 75 min, CaF_2_ was extensively removed and the Ca 2p peak shifted almost back to the peak position of enamel. Even though quantitative depth profiling was not the focus of this study, the result was in rough agreement with the nanometer-thick CaF_2_ formed on the enamel surface treated by acidic Olaflur agent done by Muller *et al.*^25^, instead of a micrometer-thick CaF_2_ reported earlier^7^. The absence of CaF_2_ formed on the enamel surface treated by neutral NaF agent (pH 6.2) reported in Muller *et al.*’s study may be due to the short exposure time (5 min) they used.

In addition, compared with the absence of the F 1s signal up to the fluoride strip application for 2 hr (Fig. 2(B)) in which fluoride was actually present, the detection of a significant F 1s peak intensity at ion etching of 150 min (Fig. 3(B)) revealed that the application had resulted in F^−^ infiltration into a depth equivalent to far more than 150-min ion etching and the apatite in the depth equivalent to ion etching between 75 and more than 150 min was mainly comprised of EFHAP/FAP (Fig. 6). The large F/Ca ratio of 0.3 observed in Fig. 4(A) in the depth equivalent to more than 75-min ion etching revealed that FAP (Ca_10_(PO_4_)_6_F_2_, in which F/Ca was 0.2), instead of EFHAP (in which F/Ca <0.2), was the main form of apatite present beneath the CaF_2_ layer. The almost unvaried F/Ca ratio with an etching time after 90 min, instead of a slowly decreasing ratio, suggested that the CaF_2_/FAP layer formed underneath the CaF_2_ layer was very thick. The thick CaF_2_/FAP layer, in addition to the CaF_2_ layer, formed on the treated enamel surface thus greatly favored the use of fluoride strips over toothpaste against enamel demineralization. Results of the IC assays of calcium ions demonstrated the favor, which showed that, for the same treatment time, the fluoride-strip-treated specimen consistently exhibited significantly higher mineral uptake and less mineral loss than the toothpaste-treated specimen (Table 1).

For the toothpaste group, the observed Ca 2p peak shift with ion etching time to higher binding energy (Fig. 3(C)) was consistent with the removal of SHAP from the toothpaste-treated specimen by the ion beam. After etching for 75 min, SHAP was extensively removed and the Ca 2p peak shifted almost back to the peak position of enamel. Compared with the large F 1s peak observed at the ion etching time between 75 and 150 min for the fluoride-strip-treated specimen (Fig. 3(B)), the 45-min survival of the F 1s signal for the toothpaste-treated specimen (Fig. 3(D)) indicated that the FHAP layer formed via the application of toothpaste was far thinner than that formed via the application of the fluoride strip. The drastic decrease in F/Ca ratio to ~0 within 5 min of ion etching on the toothpaste-treated specimen with the ratio becoming almost unvaried with etching time thereafter confirmed that F^−^ only reached a shallow depth in the toothpaste-treated specimen.

The present study has demonstrated significantly higher fluoride content present in the specimen exposed to the fluoride strip than that exposed to toothpaste. Depth profiling and surface analysis of enamel after 24 h treatment with the fluoride strip demonstrated that the outer surface of the treated enamel was covered with a CaF_2_ layer. FAP and CaF_2_ coexisted below the CaF_2_ layer. One hour of strip usage significantly decreased enamel dissolution and longer treatments increased this effect substantially. Caries prevention is a more important issue in public health than caries treatment. For those countries that do not use water fluoridation or not provide facile dental treatments, and for those people who fear caries treatment, the fluoride strip might be especially suitable and beneficial. Moreover, the present *in vitro* study demonstrated that the fluoride strip exhibited the potential as an effective fluoride delivery device for caries prevention. However, further clinical studies are required to confirm its efficacy in caries prevention and safety in long-term use.

## Additional Information

**How to cite this article**: Lee, B.-S. *et al.* Prevention of enamel demineralization with a novel fluoride strip: enamel surface composition and depth profile. *Sci. Rep.* **5**, 13352; doi: 10.1038/srep13352 (2015).

## Figures and Tables

**Table 1:** Mean (+/−SD) changes (losses or uptakes) in dissolved calcium ion concentration in the *in vitro* enamel demineralization assay.

	0 hr	1 hr	2 hr	4 hr	8 hr	24 hr
Loss, baseline	−4.3 ± 0.8					
Uptake, baseline	0.4 ± 0.3					
Net, baseline	−3.8 ± 0.7					
Loss, strip without F		−6.2 ± 0.9	−6.2 ± 0.9	−6.0 ± 0.8	−6.1 ± 0.8	−6.3 ± 1.0
Loss, fluoride strip		0.3 ± 0.3	0.7 ± 0.3	1.1 ± 0.7	1.6 ± 0.3	2.4 ± 0.5
Loss, toothpaste		−0.5 ± 0.2	−0.4 ± 0.9	−0.3 ± 1.1	0.1 ± 0.8	1.4 ± 0.6
Uptake, strip without F		−0.1 ± 0.1	−0.3 ± 0.3	−0.4 ± 0.2	−0.4 ± 0.2	−0.4 ± 0.2
Uptake, fluoride strip		1.1 ± 0.2	1.2 ± 0.3	2.9 ± 0.3	3.2 ± 0.4	3.5 ± 0.3
Uptake, toothpaste		0.6 ± 0.3	0.9 ± 0.2	1.5 ± 0.2	2.1 ± 0.3	2.4 ± 0.4
Net, strip without F		−6.2 ± 1.0	−6.5 ± 1.0	−6.4 ± 0.9	−6.6 ± 0.8	−6.7 ± 1.0
Net, fluoride strip		1.4 ± 0.1^a^	1.8 ± 0.2^a^	3.9 ± 0.5^b^	4.5 ± 0.7^c^	5.9 ± 0.3^d^
Net, toothpaste		−0.1 ± 0.3^e^	−0.1 ± 0.6^e^	1.2 ± 0.9^f^	2.2 ± 0.5^g^	3.6 ± 0.3^h^

Fluoride strip treatment significantly inhibited enamel demineralization. Calcium loss was the change, relative to the baseline, in the calcium content after treatment in the demineralizing solutions while calcium uptake was the change after treatment in the remineralizing solutions. The net value was the sum of calcium loss and calcium uptake.

^a–h^The net values having no statistically significant differences (n = 15) are identified with the same letter, whereas those having significant differences are represented with different letters.
